# Induction of matrix metalloproteinase-1 (MMP-1) during epidermal invasion of the stroma in human skin organ culture: keratinocyte stimulation of fibroblast MMP-1 production

**DOI:** 10.1054/bjoc.2001.2122

**Published:** 2001-11

**Authors:** S E Moon, M K Dame, D R Remick, J T Elder, J Varani

**Affiliations:** Departments of Pathology, Dermatology and Radiation Oncology, The University of Michigan Medical School, Ann Arbor, MI, 48109; Ann Arbor Veterans Affairs Hospital, Ann Arbor, MI, 4810

**Keywords:** invasion, epidermal growth factor (EGF)-receptor, EGF-receptor antagonist, interleukin-1 receptor antagonist, matrix metalloproteinase-1

## Abstract

Organ cultures of human skin were incubated for 8 days under growth factor-free conditions or exposed to 10 ng ml^−1^ of human recombinant epidermal growth factor (EGF) during the incubation period. Normal histological features were preserved in the absence of growth factor, while epithelial cells underwent a proliferative response and invaded the underlying stroma in the presence of exogenous EGF. The same concentrations of EGF that induced stromal invasion also resulted in up-regulation of matrix metalloproteinase-9 (MMP-9; 92-kD gelatinase B) in organ culture and keratinocyte monolayer culture, and expression of MMP-1 (interstitial collagenase) in organ culture and fibroblast monolayer culture. When skin organ cultures were exposed to a potent, irreversible EGF–receptor tyrosine kinase (EGF–RTK) antagonist along with EGF, abnormal histological features were reversed, and MMP-9 production was suppressed. In contrast, EGF-RKT antagonism had only a modest inhibitory effect on MMP-1 production. Culture fluid from keratinocytes grown in monolayer culture stimulated fibroblast proliferation and MMP-1 elaboration. Treatment of fibroblasts with the same EGF–RTK antagonist inhibited keratinocyte-induced fibroblast proliferation but had only a modest inhibitory effect (approximately 20% inhibition) on MMP-1 production. In contrast, treatment of dermal fibroblasts with Interleukin-1 Receptor Antagonist had no effect on keratinocyte-induced fibroblast growth but strongly inhibited MMP-1 production (greater than 70% inhibition). These data indicate that stromal invasion by epithelial cells in EGF-treated skin is associated with events occurring in both the epidermis and dermis. The direct effect of the exogenous growth factor appears to be primarily on the epidermis. Dermal events reflect, at least in part, a response to factors elaborated in the epidermis. © 2001 Cancer Research Campaign  http://www.bjcancer.com


*British Journal of Cancer* (2001) **85**(10), 1600–1605
© 2001 Cancer Research Campaign
doi: 10.1054/ bjoc.2001.2122, available online at http://www.idealibrary.com on IDEAL

http://www.bjcancer.com

### Induction of matrix metalloproteinase-1 (MMP-1) during epidermal invasion of the stroma in human skin organ culture: keratinocyte stimulation of fibroblast MMP-1 production

**SE Moon[1], MK Dame[1], DR Remick[1], JT Elder[2,3,4] and J Varani[1]**

Departments of [1]Pathology, [2]Dermatology and [3]Radiation Oncology. The University of Michigan Medical School, Ann Arbor, MI 48109; [4]Ann Arbor Veterans Affairs Hospital, Ann Arbor, MI 48109

**Summary** Organ cultures of human skin were incubated for 8 days under growth factor-free conditions or exposed to 10 ng ml$^{-1}$ of human recombinant epidermal growth factor (EGF) during the incubation period. Normal histological features were preserved in the absence of growth factor, while epithelial cells underwent a proliferative response and invaded the underlying stroma in the presence of exogenous EGF. The same concentrations of EGF that induced stromal invasion also resulted in up-regulation of matrix metalloproteinase-9 (MMP-9; 92-kD gelatinase B) in organ culture and keratinocyte monolayer culture, and expression of MMP-1 (interstitial collagenase) in organ culture and fibroblast monolayer culture. When skin organ cultures were exposed to a potent, irreversible EGF–receptor tyrosine kinase (EGF–RTK) antagonist along with EGF, abnormal histological features were reversed, and MMP-9 production was suppressed. In contrast, EGF-RKT antagonism had only a modest inhibitory effect on MMP-1 production. Culture fluid from keratinocytes grown in monolayer culture stimulated fibroblast proliferation and MMP-1 elaboration. Treatment of fibroblasts with the same EGF–RTK antagonist inhibited keratinocyte-induced fibroblast proliferation but had only a modest inhibitory effect (approximately 20% inhibition) on MMP-1 production. In contrast, treatment of dermal fibroblasts with Interleukin-1 Receptor Antagonist had no effect on keratinocyte-induced fibroblast growth but strongly inhibited MMP-1 production (greater than 70% inhibition). These data indicate that stromal invasion by epithelial cells in EGF-treated skin is associated with events occurring in both the epidermis and dermis. The direct effect of the exogenous growth factor appears to be primarily on the epidermis. Dermal events reflect, at least in part, a response to factors elaborated in the epidermis. © 2001 Cancer Research Campaign http://www.bjcancer.com

**Keywords**: invasion; epidermal growth factor (EGF)-receptor; EGF-receptor antagonist; interleukin-1 receptor antagonist; matrix metalloproteinase-1

Epithelial tumours of the skin (including both basal cell and squamous cell carcinomas) are highly invasive locally. It is not uncommon for such tumours to spread widely through the stroma, and to penetrate by direct extension into subcutaneous fat, muscle, cartilage and bone. Damage to host tissue is often extensive (Weedon, 1997; Brodland, 1998). Mechanisms of tissue invasion by epithelial tumours are not completely understood. Based on findings in a number of experimental models, it is believed that stromal invasion by epithelial tumours involves epithelial cell proliferation, induction of motility in the invasive cells and the up-regulation of tissue-destructive matrix metalloproteinases (MMPs) (Clark, 1991; Stetler-Stevenson et al, 1993; Werb, 1997; Herouy, 2001).

It is difficult to know precisely how the invasion process in experimental models mimics human tumour invasion under in situ conditions. As a novel way to address this issue, we have developed a model of stromal invasion by epithelial cells in organ-cultured human skin. This model of invasion is based on the finding that while normal histological structure and biochemical function are maintained in human skin under serum-free, growth factor-free conditions (Varani et al, 1993, 1994), exposure of skin in organ culture to exogenous EGF results in epidermal hyperplasia associated with abnormal differentiation and acantholysis in the upper epidermis, erosion of the epithelial–dermal basement membrane and penetration of epithelial cells into the stroma. In places, individual isolated epithelial cells can be seen completely surrounded by stroma (Fligiel and Varani, 1993; Zeigler et al, 1996a). Invasion in organ-cultured skin is accompanied by MMP up-regulation and activation (Fligiel and Varani, 1993; Varani et al, 1995; Zeigler et al, 1996a, 1996b), and is inhibited in the presence of tissue inhibitor of metalloproteinase-2 (TIMP-2) (Zeigler et al, 1996b).

The present study continues our efforts to delineate cellular and molecular events that contribute to invasion in this model. Here we show that keratinocyte proliferation and elaboration of MMP-9 (92-kD gelatinase B) are stimulated by EGF and inhibited (both in monolayer culture and organ culture) by a potent, irreversible pharmacological antagonist of EGF receptor tyrosine kinase (EGF-RTK). Further studies reveal that dermal fibroblast proliferation and MMP-1 (interstitial collagenase) production also occur following EGF stimulation. These events occur, at least in part, in response to factors elaborated by epidermal cells. Fibroblast proliferation is strongly inhibited by the same EGF-RTK antagonist that blocks keratinocyte proliferation. In contrast, MMP-1

*Received 2 April 2001*
*Revised 4 July 2001*
*Accepted 18 July 2001*

*Correspondence to:* J Varani production is resistant to EGF-RTK antagonism but inhibited by interleukin-1 receptor antagonism.

### MATERIALS AND METHODS

### Reagents

The EGF-RTK antagonist used in these studies was a generous gift of Drs WL Leopold and David Fry of Pfizer Global Research and Development, Ann Arbor Laboratories, Ann Arbor, MI. The compound, designated as PD169540, is an acrylamide-substituted 4-anilinopyrido[d]pyrimidine designed to irreversibly alkylate Cys-773 within the ATP-binding pocket of c-erbB1 and c-erbB2 (EGF receptor family members) (Smaill et al, 2000). Recombinant forms of human EGF, interleukin-1β (IL-1β) and IL-1 Receptor Antagonist were obtained from R&D Systems (Minneapolis, MN).

### Human skin organ cultures

Replicate 2 mm full-thickness punch biopsies of sun-protected hip skin were obtained from adult volunteers. Immediately upon biopsy, the tissue was immersed in culture medium consisting of Keratinocyte Basal Medium (KBM) (Clonetics, Inc, Walkersville, MD). KBM is a low-$Ca^{2+}$ (0.15 mM) serum-free modification of MCDB-153 medium optimized for high-density keratinocyte growth (Boyce and Ham, 1983). The culture medium was supplemented with $CaCl_2$ to bring the final $Ca^{2+}$ concentration to 1.4 mM. This was done because previous studies had shown that survival of human skin in organ culture depends on a $Ca^{2+}$ concentration that is optimized for fibroblast survival and growth (Varani et al, 1993, 1994). After transport to the laboratory on ice, the biopsies were incubated in wells of a 96-well dish containing 200 μl of $Ca^{2+}$-supplemented KBM with or without additional treatments. Cultures were incubated at 37˚C in an atmosphere of 95% air and 5% $CO_2$. Incubation was for 8 days, with change of medium containing the various treatments every 2 days. At the end of the incubation period, tissue was fixed in 10% buffered formalin and examined histologically after staining with haematoxylin and eosin. All procedures involving human subjects were approved by the University of Michigan Institutional Review Board, and all subjects provided written informed consent prior to inclusion in the study.

### Human epidermal keratinocytes in monolayer culture

Additional skin biopsies were used for isolation of keratinocytes and fibroblasts. Normal human epidermal keratinocytes were obtained as described previously (Varani et al, 1994) and maintained in monolayer culture using Keratinocyte Growth Medium (KGM) (Clonetics, Inc). KGM contains the same basal medium as KBM, but is further supplemented with a mixture of growth factors including 0.1 ng $ml^{-1}$ EGF, 0.5 μg $ml^{-1}$ insulin, and 2% bovine pituitary extract. Growth was at 37˚C in an atmosphere of 95% air and 5% $CO_2$. Cells were sub-cultured by exposure to trypsin/EDTA and used at passage 2–3. In some experiments, the HaCaT line of immortalized human epidermal keratinocytes (Boukamp et al, 1988) was used in place of normal keratinocytes. HaCaT cells were propagated in exactly the same manner as low-passage keratinocytes, and used interchangeably with keratinocytes.

Culture fluid was prepared from keratinocyte or HaCaT cultures as follows. The cells were plated at $4 \times 10^4$ cells $cm^{-2}$ of surface area in 25 $cm^2$ or 75 $cm^2$ flasks. KGM was used as culture medium. When the cultures were approximately 75% confluent, the cells were washed 2 times in KBM and incubated for 72 hours in KBM supplemented with 1.4 mM $Ca^{2+}$. At the end of the incubation period, the culture fluid was collected and separated from cells and debris by low-speed centrifugation. The keratinocyte culture fluid was used as a source of ‘stimulating factor(s)’ for dermal fibroblast proliferation and MMP-1 production.

### Human dermal fibroblasts in monolayer culture

Normal human dermal fibroblasts were isolated as described previously (Varani et al, 1994) and grown in monolayer culture using Dulbecco’s Modified Minimal Essential Medium supplemented with non-essential amino acids and 10% fetal bovine serum (DMEM-FBS) as culture medium. Fibroblasts were maintained at 37˚C in an atmosphere of 95% air and 5% $CO_2$. Cells were sub-cultured by exposure to trypsin/EDTA and used at passage 2–5.

For proliferation assays, dermal fibroblasts were plated at $2 \times 10^4$ cells $cm^{-2}$ of surface area in wells of a 24-well dish using DMEM-FBS as growth medium. After allowing cells to attach, the medium was removed and the cells washed twice in $Ca^{2+}$-supplemented KBM. The cells were then incubated for 72 hours in $Ca^{2+}$-supplemented KBM with or without keratinocyte culture fluid and/or additional reagents as indicated in Results. At the end of the incubation period, the cells were harvested by trypsinization and enumerated using an automated particle counter. Culture fluid was obtained and clarified by low-speed centrifugation, following which it was assayed for MMP-1 as indicated below.

### Substrate-embedded enzymography

SDS-PAGE substrate-embedded enzymography (zymography) was used to identify enzymes with collagenase and gelatinase activities. Assays were carried out exactly as described in a previous report (Gibbs et al, 1999). Briefly, denatured but non-reduced culture fluid samples were resolved in 7.5% SDS-PAGE gels prepared with the added incorporation of gelatin (1 mg $ml^{-1}$) or β-casein (1 mg $ml^{-1}$) prior to casting. After electrophoresis, gels were washed twice for 15 min in 50 mM Tris buffer containing 1 mM $Ca^{2+}$, 0.5 mM $Zn^{2+}$ and 2.5% Triton X-100. The gels were then incubated overnight in Tris buffer with 1% Triton X-100 and stained the following day with Coumassie Brilliant Blue 250-R. Following destaining, zones of enzyme activity were detected as regions of negative staining against the dark background. The zymograms were converted to negative images and digitized. Quantitation was accomplished by determining the number of pixels in the negative images. For organ cultures, data were normalized per biopsy. For cell cultures, data were normalized to an equivalent number of cells. Volumes of 5–35 μl of undiluted organ culture or cell culture fluid were normally used for these assays; zones of activity were proportional to the quantity of culture fluid used. Gelatin zymography is useful for detection of MMP-2 (72-kD gelatinase A) and MMP-9. β-casein zymography is useful for detection of MMP-1, which appears as a doublet in the 54-kD region of the gel. The β-casein zymographic bands co-migrate with purified MMP-1 as detected in Western blotting

© 2001 Cancer Research Campaign

(Varani et al, 2000). Digestion of native, fibrillar type I collagen was observed in parallel with expression of MMP-1, and blocked in the presence of TIMP-2 or EDTA but not with a battery of serine proteinase inhibitors (Varani et al, 2000).

### RESULTS

### Effects of EGF-RTK antagonism on proliferation and abnormal differentiation induced by EGF in organ-cultured skin

In the first series of experiments, organ cultures of adult human skin were incubated for 8 days in serum-free, growth factor-free culture medium (i.e., KBM containing 1.4 mM $Ca^{2+}$) or in the same medium supplemented with 10 nM EGF and/or 1 μM EGF-RTK antagonist. As shown in Figure 1, normal histological architecture was preserved in control tissue. Consistent with previous reports (Varani et al, 1993, 1994), the histological appearance of the tissue resembled that of freshly biopsied skin. Basal epithelial cells were mainly columnar in shape, with orderly stratification of suprabasal keratinocytes. In the absence of exogenous EGF, the irreversible EGF-RTK antagonist at concentrations as high as 1 μM had no demonstrable effect on histology (not shown).

The appearance of EGF-treated tissue was significantly different from that of control tissue (Figure 1). The epidermis was thickened, due to an increase in the number of epidermal keratinocytes. Routinely, epidermal cell down-growth into stromal space was observed, and in places, isolated epithelial cells could be seen surrounded by stroma. The epidermis of EGF-treated tissue was more disorganized than that of control tissue, with loss of the normal progression from basal to spinous to granular layers. Loss of suprabasal keratinocyte cohesion was frequently observed, leading to acantholysis and partial or complete separation of the upper spinous layers in many of the EGF-treated specimens. These histological changes observed in the presence of exogenous EGF are consistent with what has been described in previous reports (Fligiel and Varani, 1993; Zeigler et al, 1996a, 1996b).

Figure 1 also demonstrates the histological appearance of human skin after 8 days in organ culture in the presence of exogenous EGF and the irreversible EGF-RTK antagonist. It can be seen that the antagonist reversed the effects of 10 nM EGF at the concentration used (1 μM). The histological appearance of organ cultures treated with EGF + PD169540 was very similar to that of control organ cultures not treated with either reagent. It has been established previously that this concentration of PD169540 is without effect on tyrosine kinases residing outside the EGF receptor family (Smaill et al, 2000). The effect of the antagonist was dose-dependent: 0.5 μM PD169540 was also effective, albeit less so than 1 μM, whereas lower concentrations had no effect (data not shown).

### Effects of EGF-RTK antagonism on MMP-1 and MMP-9 production in organ-cultured skin

Culture fluids were obtained from control organ-cultures (i.e., from tissue incubated in KBM supplemented with 1.4 mM $Ca^{2+}$) and from cultures treated with 10 ng $ml^{-1}$ EGF in the absence or presence of the EGF-RTK antagonist. Exposure of the skin in organ culture to EGF resulted in up-regulation of MMP-1 and MMP-9 without a major change in MMP-2 expression (Figure 2). Consistent with past findings (Varani et al, 1995; Zeigler et al, 1996b), MMP-9 up-regulation occurred rapidly (evident within 2 days and maximal by day 4). MMP-1 up-regulation occurred

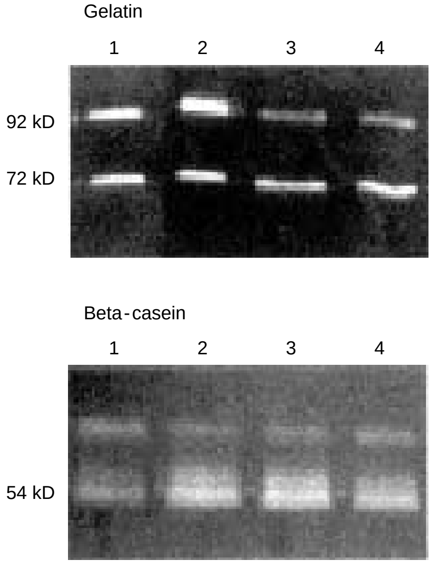

**Figure 2** EGF-RTK antagonism inhibits MMP-9 and MMP-1 up-regulation in EGF-treated, organ-cultured skin. Human skin was maintained in organ culture for 8 days. Organ culture fluid was collected at 2-day intervals and assessed by gelatin and β-casein zymography. **Upper panel**: Gelatin zymography (day 4): MMP-9 (gelatinase B) is observed at the 92-kD locus while MMP-2 (gelatinase A) is seen at the 72-kD locus. **Lower panel**: β-casein zymography (day 6): MMP-1 (interstitial collagenase) is seen as a doublet at the 54-kD locus. Lane 1, KBM + $Ca^{2+}$; lane 2, KBM + $Ca^{2+}$ + 10 ng $ml^{-1}$ EGF, lanes 3 and 4, same as lane 2 except with 0.5 and 1.0 μM PD169540, respectively. The findings depicted here were consistent with organ cultures from 5 different individuals

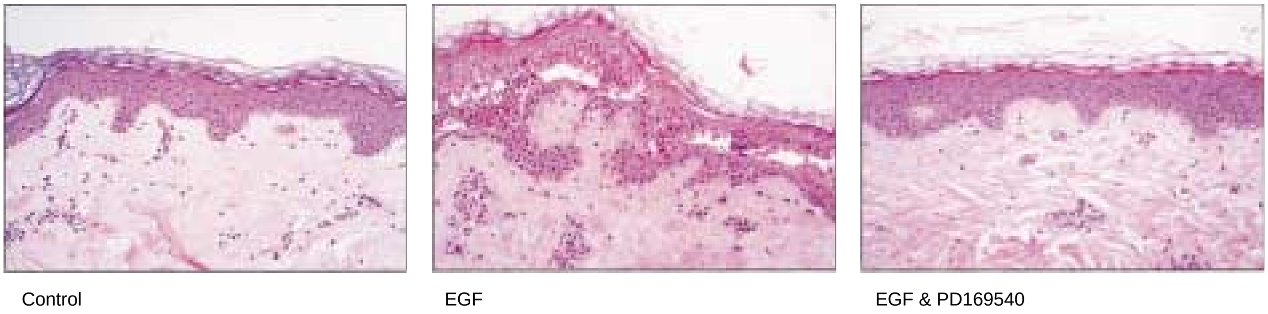

**Figure 1** EGF-RTK antagonism reverses histological features associated with stromal invasion in EGF-treated, organ-cultured skin. Human skin was maintained in organ culture for 8 days. At the end of the incubation period, tissue was stained with haematoxylin and eosin. The histological appearances depicted in the 3 panels were consistent among skin organ cultures from 8 different individuals

*© 2001 Cancer Research Campaign*

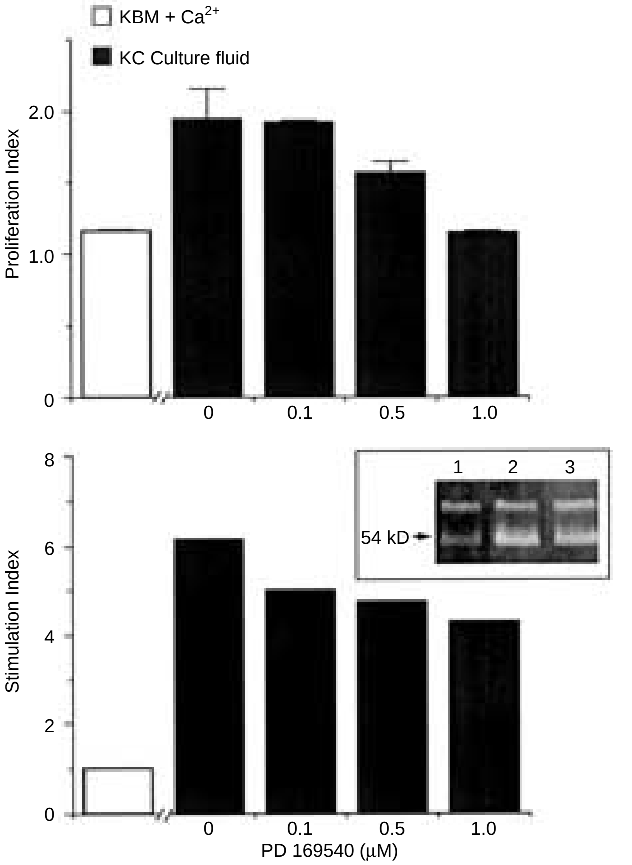

**Figure 3** EGF-RTK antagonism inhibits human dermal fibroblast proliferation and MMP-1 production in response to culture fluid from human epidermal keratinocytes. **Upper panel**: Proliferation: Values are presented as proliferation indices; i.e., the ratio of cells present at the end of the 48 hour incubation period to cells present at time-zero ± standard deviations in a single experiment. The experiment was repeated 4 times with similar results. **Lower panel**: MMP-1 production: Values shown represent densitometry scans of the 54-kD bands in β-casein gels, and quantification of the digitized scans. Values are presented as stimulation indices; i.e., the ratio of pixels in each of the treatment groups (closed bars) to pixels in the control group (open bar). The experiment was repeated 4 times with similar results. The insert demonstrates 54-kD β-caseinolytic activity elaborated by control fibroblasts (lane 1), fibroblasts exposed to keratinocyte culture fluid (lane 2) or fibroblasts exposed to 1 μM PD169540 along with keratinocyte culture fluid (lane 3)

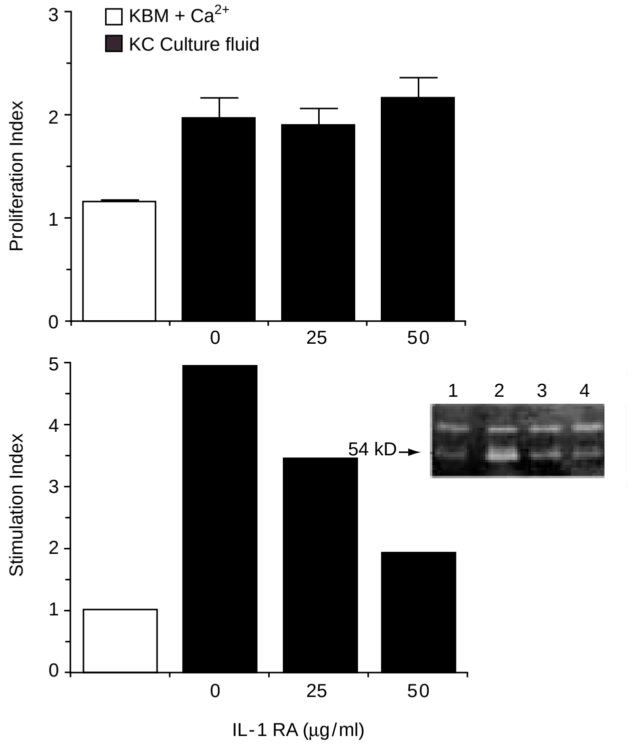

**Figure 4** IL-1 receptor antagonism fails to inhibit fibroblast proliferation in monolayer culture induced by culture fluid from human epidermal keratinocytes but inhibits MMP-1 up-regulation induced in the same cells. **Upper panel**: Proliferation assay. Values are presented as proliferation indices; i.e., the ratio of cells present at the end of the 48 hour incubation period to cells present at time-zero ± standard deviations in a single experiment. The experiment was repeated 4 times with similar results. **Lower panel**: MMP-1 up-regulation. Values shown represent densitometry scans of the 54-kD bands in β-casein gels, and quantification of the digitized scans. Values are presented as stimulation indices; i.e., the ratio of pixels in each of the treatment groups (closed bars) to pixels in the control group (open bar). The experiment was repeated four times with similar results. The insert demonstrates 54-kD β-caseinolytic activity elaborated by control fibroblasts (lane 1), fibroblasts exposed to keratinocyte culture fluid (lane 2) or fibroblasts exposed to 25 or 50 μg $ml^{-1}$ of the IL-1 receptor antagonist (IL-1 RA) along with keratinocyte culture fluid (lanes 3 and 4).

more slowly; differences between control and EGF-treated tissue were not routinely observed until day 6–8. In the presence of PD169540, MMP-9 production was substantially reduced without a detectable change in MMP-2 (Figure 2, upper panel). The concentrations of antagonist that inhibited enzyme induction (0.5 – 1.0 μM) were the same concentrations that also blocked epidermal proliferation, abnormal differentiation and stromal invasion. MMP-1 elaboration was also inhibited in the presence of PD169540 (Figure 2, lower panel). In contrast to MMP-9, where production was inhibited to below basal levels, MMP-1 was inhibited only modestly and only at the highest concentration of antagonist.

### Effects of EGF-RTK antagonism on fibroblast proliferation and fibroblast production of MMP-1 in response to keratinocyte-derived factors

Based on previous studies in several laboratories including our own (Varani et al, 1995; Fisher et al, 1997; Varani et al, 2000), it is accepted that the epidermis is the major source of MMP-9 in the skin. It is further known that up-regulation of MMP-9 in keratinocytes following stimulation with growth factors (including EGF) is a direct consequence of signalling through mitogen-activated protein (MAP) kinase cascades leading to formation of the AP-1 transcription complex and MMP-9 gene transcription (Zeigler et al, 1999). Although epidermal keratinocytes are also capable of synthesizing MMP-1 (Bauer et al, 1977; Goslen and Bauer, 1986; Pilcher et al, 1999), dermal fibroblasts appear to be the major source of this enzyme in skin (Fisher et al, 1997). The sequence of events leading to induction of MMP-1 in the dermis of growth factor-treated skin is not fully understood. Here we present evidence that factors released from keratinocytes are able to stimulate MMP-1 production (as well as growth) in dermal fibroblasts.

In the presence of keratinocyte-conditioned culture fluid (500 μl of culture fluid per ml of medium), fibroblast growth and MMP-1 production were both stimulated (Figure 3). Additional studies demonstrated that concentrations of keratinocyte culture fluid as low as 50–100 μl $ml^{-1}$ were effective (not shown). It can be seen from Figure 3 (upper panel) that the same concentrations of PD169540 that inhibited invasion in organ culture (0.5 – 1.0 μM, see above) also suppressed keratinocyte-induced fibroblast growth (greater than 95% inhibition). PD169540 also inhibited fibroblast production of MMP-1 in response to keratinocyte culture fluid.

*© 2001 Cancer Research Campaign*

In contrast to its effects on proliferation, however, only modest inhibition of MMP-1 production (approximately 20%) was seen (Figure 3, lower panel). Control studies carried out in parallel demonstrated that fibroblast proliferation and MMP-1 production induced by exogenous EGF (0.1 – 10 ng $ml^{-1}$) were both completely inhibited in the presence of the EGF-RTK antagonist (not shown).

Since EGF-RTK antagonism was only modestly effective in preventing the induction of MMP-1 by keratinocyte culture fluid, additional studies were conducted in which fibroblasts were exposed to the same culture fluid and concomitantly treated with human recombinant IL-1 Receptor Antagonist. In the presence of the IL-1 Receptor Antagonist (25– 50 μg $ml^{-1}$), keratinocyte culture fluid stimulation of fibroblast proliferation was unaffected, while MMP-1 production was reduced by approximately 70–80% (Figure 4). Additional studies showed that concentrations of IL-1 Receptor antagonist as low as 10 μg $ml^{-1}$ were effective in inhibiting MMP-1 up-regulation. Not surprisingly, the IL-1 Receptor Antagonist also prevented MMP-1 induction by exogenous IL-1β, but had no effect on EGF-stimulated MMP-1 production (not shown).

### DISCUSSION

Invasion of the stroma by epithelial cells in growth factor-treated human skin is associated with keratinocyte proliferation and up-regulation of MMP-9 (Fligiel and Varani, 1993; Varani et al, 1995; Zeigler et al, 1996a, 1996b). Exposure of epidermal keratinocytes in monolayer culture to the same growth factors duplicates these effects on proliferation and MMP-9 production (Varani et al, 1995; Zeigler et al, 1996a, 1996b). The present studies demonstrate that blocking EGF-RTK activity inhibits epidermal proliferation and MMP-9 production in organ culture and monolayer culture, and concomitantly prevents stromal invasion in organ culture. Taken together, these in vitro and in vivo findings strongly suggest that invasion-associated events in the epidermis occur as a direct consequence of EGF receptor activation on the keratinocyte surface.

Although events occurring in the epidermis are, undoubtedly, critical for stromal invasion by epithelial cells, it is likely that events occurring in the dermis are important as well. Specifically, the destruction of stromal connective tissue by collagenolytic enzymes is an important feature of epithelial tumour invasion. This is particularly important in the skin, where the stroma consists of a thick, dense collagenous matrix (Bauer et al, 1977; Goslen and Bauer, 1986; Weedon, 1997; Brodland, 1998). Although there are at least 3 mammalian collagenolytic enzymes that can mediate destruction of type I collagen, our recent studies suggest that MMP-1 is the major enzyme involved in this process (Varani et al, 2000). Other recent data also suggest that although epithelial cells can elaborate collagenolytic enzymes including MMP-1 (Pilcher et al, 1999), the major source of these enzymes is the dermis (Fisher et al, 1997; Varani et al, 2000). Here we show that regulation of MMP-1 production in growth factor-treated skin is a complex process. In part, MMP-1 up-regulation may reflect a direct response of dermal fibroblasts to the exogenously added EGF. Additionally, however, it appears that factors elaborated by epidermal keratinocytes play a role in stimulating dermal fibroblasts to produce MMP-1. This is based on the finding that culture fluid from keratinocytes grown in monolayer culture was able to stimulate dermal fibroblasts to produce MMP-1. Under the culture conditions in which this occurred, there was no source of exogenous growth factor. In addition, the time-course for up-regulation of MMP-1 in organ culture (slower than that for MMP-9) is consistent with an indirect response to the exogenous stimulus.

The nature of the keratinocyte-derived factors that influence fibroblast MMP-1 production remains to be elucidated. The data suggest that there are at least two factors – one an EGF receptor agonist and the other an EGF receptor-independent factor. Based on inhibition data with PD169540, it appears that the EGF receptor activation plays a relatively minor role in up-regulating fibroblast MMP-1 production. In contrast, factors acting through the EGF receptor are more important for stimulating fibroblast growth. Keratinocytes are known to elaborate a number of molecules that can activate EGF receptors, including amphiregulin, transforming growth factor-α and a heparin-binding EGF-like molecule (HB-EGF) (Elder et al, 1990; Elder, 1994; Stoll et al, 1997). At this point, we have no way of knowing which of these or other possible ligands is critical. It should be noted in this regard, however, that of the known keratinocyte-derived ligands for the EGF receptor, only HB-EGF is strongly up-regulated in culture (Stoll et al, 1997).

Since MMP-1 production in organ culture (as well as in fibroblasts exposed to keratinocyte culture fluid in monolayer culture) was only modestly sensitive to EGF-RTK antagonism, ligands unrelated to the EGF receptor system are likely to be important as stimulators of this enzyme. IL-1 is one candidate. Both IL-1α and IL-1β are potent inducers of MMP-1 production in dermal fibroblasts (Mackay et al, 1992; Shingu et al, 1993) and our own unpublished data as well as published reports (Maas-Szabowski et al, 1999, 2000) indicate that the amount of IL-1 elaborated by keratinocytes in culture (estimated to be up to 450 pg of IL-1α and 100 pg of IL-1β per $1 \times 10^6$ cells in a 2-day period) is sufficient to elicit the fibroblast response. Whether IL-1 is the only non-EGF receptor ligand involved is not known, but the fact that substantial inhibition (70–80%) of MMP-1 up-regulation was achieved with IL-1 Receptor Antagonist suggests that it may be. It is of interest in this regard that MMP-1 production in keratinocytes may be regulated much differently. It was shown in a recent study by Wan et al (2001) that keratinocyte production of MMP-1 in response to IL-1β treatment was sensitive to inhibitors of EGF receptor phosphorylation and MAP kinase signalling. The conclusion drawn from this was that in epidermal keratinocytes, IL-1β induces MMP-1 production via EGF receptor transactivation.

The phenotype of organ-cultured skin following exposure to exogenous growth factor treatment (present report and references Fligiel and Varani, 1993; Varani et al, 1995; Zeigler et al, 1996a, 1996b, 1999) closely mimics the phenotype expressed in actual epithelial tumours (Weedon, 1997; Brodland, 1998). The proximal causes of the phenotypic abnormalities may also be similar in growth factor-treated normal skin and skin tumours. A variety of defects in EGF signalling pathways have been described in malignant epithelial tumours, including over-expression of ligands for EGF receptors, over-expression of cell surface EGF receptors and molecular abnormalities in receptor structure, leading to auto-activation (Stoscheck and King, 1986; Gottlieb et al, 1988; Ogiso et al, 1988; Wells, 1999). These defects are thought to underlie enhanced signalling through MAP kinase pathways with the attendant biological consequences (increased proliferation and MMP production) that follow. In the same manner, abnormalities in EGF signalling in malignant epithelial tumours has been shown to result in enhanced expression of factors such as IL-1 that influence

© 2001 Cancer Research Campaign dermal functions (Mass-Szabowski et al, 1999, 2000). It, thus, might be concluded that by exposing organ-cultured skin to high levels of exogenous EGF, we not only induce phenotypic changes which mimic those expressed by epithelial tumours during invasion, but also duplicate the abnormal signalling events that constitute the proximal cause of the abnormal phenotype.

### ACKNOWLEDGEMENTS

This study was supported in part by grants CA60958, DK59169 and GM50401 from the USPHS and by a grant from Pfizer Global Research and Development.

### REFERENCES

Bauer EA, Gordon JM, Reddick ME and Eisen AZ (1977) Quantitation and immunochemical localization of human skin collagenase in basal cell carcinoma. *J Invest Dermatol* **69**: 363–367

Boukamp P, Petrussevska RT, Breitkreutz D, Hornung J, Markham A and Fusenig NE (1988) Normal keratinization in a spontaneously immortalized aneuploid human keratinocyte cell line. *J Cell Biol* **106**: 761–771

Boyce S and Ham R (1983) Normal human epidermal keratinocytes. In: Webber M and Sekely L (eds) *In Vitro Models for Cancer Research* p 245–274 CRC Press: Boca Raton, FL

Brodland DG (1998) Basal Cell Carcinoma: Features associated with metastasis In: *Cutaneous Oncology: Pathophysiology, Diagnosis and Management*. SJ Miller and ME Maloney (eds.). Blackwell Science, Inc. Malden MA pp 657–663

Clark W (1991) Tumor progression and the nature of cancer. *Brit J Cancer* **64**: 631–644

Elder JT, Tavakkol A, Klein SB, Zeigler ME, Wicha M and Voorhees JJ (1990) Protooncogene expression in normal and psoriatic skin. *J Invest Dermatol* **94**: 19–25

Elder JT (1994) Transforming growth factor-alpha and related growth factors. In: *Epidermal Growth Factors and Cytokines*. T Luger and T Schwarz (editors), Marcel Dekker Inc, New York, 205–240

Fisher GJ, Wang Z-Q, Datta S, Varani J and Voorhees JJ (1997) Pathophysiology and retinoic acid prevention of sun-induced premature skin aging. *New Eng J Med* **337**: 1419–1428

Fligiel SEG and Varani J (1993) *In situ* epithelial cell invasion in organ culture. *Invasion & Metastasis* **13**: 225–233

Gibbs DF, Apel IJ, Warner RO, Weiss SJ, Johnson KJ and Varani J (1999) Characterization of matrix metalloproteinases produced by rat alveolar macrophages. *Amer J Respir Cell Molecular Biol* **20**: 1136–1144

Goslen JB and Bauer EA (1986) Basal cell carcinoma and collagenase. *J Dermatol Surg Oncol* **12**: 812–817

Gottlieb AB, Chang CK, Posnett ND, Fanelli B and Tam JP (1988) Detection of TGF-a in normal, malignant and hyper-proliferative human keratinocytes. *J Exp Med* **167**: 670–675

Herouy Y (2001) Matrix metalloproteinases in skin pathology. *Int J Molec Medicine* **7**: 3–12

Maas-Szabowski N, Shimotoyodome A and Fusenig NE (1999) Keratinocyte growth regulation in fibroblast cocultures via a double paracrine mechanism. *J Cell Sci* **112**: 1843–1853

Maas-Szabowski N, Stark H-J and Fusenig NE (2000) Keratinocyte growth regulation in defined organotypic cultures through IL-1-induced keratinocyte growth factor expression in resting fibroblasts. *J Invest Dermatol* **114**: 1075–1084

Mackay AR, Ballin M, Pelina M, Farina AR, Nason AM, Hartzler JL and Thorgeirsson UP (1992) Effect of phorbol ester and cytokines on matrix metalloproteinase and tissue inhibitor of metalloproteinase expression in tumor and normal cell lines. *Invasion & Metastasis* **12**: 168–184

Ogiso Y, Oikawa T, Kondo N, Kuzumaki N, Sugihara T and Ohura T (1988) Expression of proto-oncogenes in normal and tumor tissue of human skin. *J Invest Dermatol* **90**: 841–844

Pilcher BK, Dumin JA, Schwartz MJ, Mast BA, Schultz GS, Parks WC and Welgus HG (1999) Keratinocyte collagenase-1 expression requires an epidermal growth factor receptor autocrine mechanism. *J Biol Chem* **274**: 10372–10381

Shingu M, Nagai Y, Isayama T, Naono T, Nobunaga M and Nagai Y (1993) The effects of cytokines on metalloproteinase inhibitors (TIMP) and collagenase production by human chondorcytes and TIMP production by synovial cells and endothelial cells. *Clin Exp Immunol* **94**: 145–149

Smaill JB, Rewcastle GW, Loo JA, Greis KD, Chan OH, Reyner EL, Lipka E, Showalter HD, Vincent PW, Elliott WL and Denny WA (2000) Tyrosine kinase inhibitors. 17. Irreversible inhibitors of the epidermal growth factor receptor: 4-(phenylamino)quinazoline-and 4-(phenylamino)pyrido[3,2-d] pyrimidine-6-acrylamides bearing additional solubilizing functions. *J Med Chem* **43**: 1380–1397

Stetler-Stevenson WG, Aznavoorian S and Liotta LA (1993) Tumor cell interactions with the extracellular matrix during invasion and metastassis. *Ann Rev Cell Biol* **9**: 541–573

Stoll S, Garner W and Elder JT (1997) Heparin-binding ligands mediate autrocrine epidermal growth factor receptor activation in skin organ culture. *J Clin Invest* **100**: 1271–1281

Stoscheck CM and King LE jr (1986) Functional and structural characteristics of EGF and its receptor and their relationship to transforming proteins. *J Cell Biochem* **31**: 135–152

Varani J, Fligiel SEG, Schuger L, Perone P, Inman DR, Griffiths CEM and Voorhees JJ (1993) Effects of all-trans retinoic acid and $Ca^{2+}$ on human skin in organ culture. *Am J Pathology* **142**: 189–198

Varani J, Perone P, Griffith CEM, Inman DR, Fligiel SEG and Voorhees JJ (1994) All-trans-retinoic acid (RA) stimulates events in organ cultured human skin that underlie repair. *J Clin Invest* **94**: 1747–1756

Varani J, Perone P, Inman DR, Burmeister W, Schollenberger SB, Sitrin RJ and Johnson KJ (1995) Human skin in organ culture: Elaboration of proteolytic enzymes under growth factor-free and growth factor-containing conditions. *Amer J Pathol* **146**: 210–217

Varani J, Hattori Y, Chi Y, Schmidt T, Perone P, Zeigler ME, Fader DJ and Johnson TM (2000) Collagenolytic and gelatinolytic matrix metalloproteinases and their inhibitors in basal cell carcinoma of skin: Comparison with normal skin. *Brit J Cancer* **82**: 657–665

Wan YS, Belt A, Wang ZQ, Voorhees JJ and Fisher G (2001) Transmodulation of epidermal growth factor receptor mediates IL-1 beta-induced MMP-1 expression in human keratinocytes. *International J Molec Medicine* **7**: 329–334

Weedon D (1997) *Skin Pathology.* Churchill Livingstone, New York. pp 635–671

Wells A (1999) EGF Receptor. *International J Biochem Cell Biol* **31**: 637–643

Werb Z (1997) ECM and cell surface proteolysis: regulating cellular ecology. *Cell* **91**: 439–442

Zeigler ME, Krause S, Karmiol S and Varani J (1996a) Growth factor-induced epidermal invasion of the dermis in human skin organ culture: Dermal invasion correlated with epithelial cell motility. *Invasion & Metastasis* **16**: 3–10

Zeigler ME, Dutcheshen NT, Gibbs DF and Varani J (1996b) Growth factor-induced epidermal invasion of the dermis in human skin organ culture: Expression and role of matrix metalloproteinases. *Invasion & Metastasis* **16**: 11–18

Zeigler ME, Chi Y, Schmidt T and Varani J (1999) Role of ERK and JNK pathways in regulating cell motility and matrix metalloproteinase 9 production in growth factor-stimulated human epidermal keratinocytes. *J Cell Physiol* **180**: 271–284

© 2001 Cancer Research Campaign
